# Function of the RNA Coliphage Qβ Proteins in Medical In Vitro Evolution

**DOI:** 10.3390/mps1020018

**Published:** 2018-05-31

**Authors:** Rana L. Singleton, Carrie A. Sanders, Kevin Jones, Bobby Thorington, Timothy Egbo, Mamie T. Coats, Alain Bopda Waffo

**Affiliations:** 1 Department of Biological Sciences, College STEM, 1627 Hall Street, Montgomery, AL 36101, USA; rls0055@tigermail.auburn.edu (R.L.S.); carriec1c3@gmail.com (C.A.S.); kmj2@ymail.com (K.J.); bobbythorington@yahoo.com (B.T.); egboty@gmail.com (T.E.); mcoats@alasu.edu (M.T.C.); 2 Center for NanoBiotechnology Research, 1627 Harris Way, Montgomery, AL 36104, USA

**Keywords:** Qβ, read-through protein A_1_, foot-and-mouth disease virus (FMDV), membrane proximal external region (MPER), human immunodeficiency virus (HIV), in vitro evolution, proofreading

## Abstract

Qβ is a positive (+) single-stranded RNA bacteriophage covered by a 25 nm icosahedral shell. Qβ belongs to the family of Leviviridae and is found throughout the world (bacterial isolates and sewage). The genome of Qβ is about 4.2 kb, coding for four proteins. This genome is surrounded by 180 copies of coat proteins (capsomers) each comprised of 132 residues of amino acids. The other proteins, the subunit II (β) of a replicase, the maturation protein (A_2_) and the read-through or minor coat protein (A_1_), play a key role in phage infection. With the replicase protein, which lacks proofreading activity, as well as its short replication time, and high population size, Qβ phage has attractive features for in vitro evolution. The A_1_ protein gene shares the same initiation codon with the coat protein gene and is produced during translation when the coat protein’s UGA stop codon triplet (about 400 nucleotides from the initiation) is suppressed by a low level of ribosome misincorporation of tryptophan. Thus, A_1_ is termed the read-through protein. This RNA phage platform technology not only serves to display foreign peptides but is also exceptionally suited to address questions about in vitro evolution. The C-terminus of A_1_ protein confers to this RNA phage platform an exceptional feature of not only a linker for foreign peptide to be displayed also a model for evolution. This platform was used to present a peptide library of the G-H loop of the capsid region P1 of the foot-and-mouth disease virus (FMDV) called VP1 protein. The library was exposed on the exterior surface of Qβ phages, evolved and selected with the monoclonal antibodies (mAbs) SD6 of the FMDV. These hybrid phages could principally be good candidates for FMDV vaccine development. Separately, the membrane proximal external region (MPER) of human immunodeficiency virus type 1 (HIV-1) epitopes was fused with the A_1_ proteins and exposed on the Qβ phage exterior surface. The engineered phages with MPER epitopes were recognized by anti-MPER specific antibodies. This system could be used to overcome the challenge of effective presentation of MPER to the immune system. A key portion of this linear epitope could be randomized and evolved with the Qβ system. Overall, antigens and epitopes of RNA viruses relevant to public health can be randomized, evolved and selected in pools using the proposed Qβ model to overcome their plasticity and the challenge of vaccine development. Major epitopes of a particular virus can be engineered or displayed on the Qβ phage surface and used for vaccine efficacy evaluation, thus avoiding the use of live viruses.

## 1. Introduction

Viruses with RNA genomes are major causes of emerging deadly infectious diseases and represent the most ever-present and everlasting changing cellular parasites known. There are many different alterations of genetic elements occurring during RNA genome replication [1,2]. This plasticity is fundamental and caused by infidelity of the replication process [3,4]. Evolution of RNA viruses is basically unpredictable due to the stochastic nature of the mutation and recombination events, as well as environmental factors. A fundamental concern regarding RNA viruses is their high mutation rate [2,5]. RNA viruses exist as quasispecies which are the result of pressure, mutation, and selection, in ways which are poorly understood. These mutations could affect the binding capability of the virus to both neutralizing antibodies and host receptors which could result in emergence of new viral host ranges [6]. The chance that defined molecules may promote selection of escape mutants or a particular detection tool may fail to detect the virus is high.

The mechanisms of viral infection and prevention are the most difficult investigations in virology research. Virus tropism and neutralizing antibodies are the major crucial steps in virus life cycles and vaccine development respectively. To better understand the molecular pathways of infection and prevention, we must utilize synthetic biology. Synthetic biologists would simplify and engineer complex artificial biological systems that investigate natural biological phenomena [7,8].

The current generation of synthetic vaccines, diagnostic reagents (monoclonal antibodies (mAbs), peptide antigens, oligonucleotides, etc.) and therapeutics may be very adequate to prevent, detect and cure infections due to DNA viruses. Unfortunately, these fail to address RNA viruses. This is largely due to the vast number of circulating quasispecies associated with RNA pathogens. The dynamics of viral quasispecies mandates careful consideration. In reality, prevention and therapy of infection due to an RNA virus should rely on multicomponent vaccines and antiviral agents that address the complexity of the RNA quasispecies mutant spectra. This can be accomplished using the RNA Qβ coliphage display peptide library.

Phage display technology has been developed, applied extensively and proven to have success in exposing functional peptides on the exterior surface of phage [9]. A novel display system which exploits the high mutation rate of RNA replicase has been developed [10,11]. A small peptide library has been presented on this RNA phage surface and selected against the SD6 mAbs of the foot-and-mouth disease virus (FMDV). The membrane-proximal external region (MPER) of the human immunodeficiency virus type-1 (HIV-1) was engineered on the surface of Qβ as well. These studies use the Qβ phage for its large population and high mutation rate [12]. The novel pipeline proposed herein takes into consideration the quasispecies nature of these pathogenic RNA viruses. This review will present a detailed structural and functional synopsis of the Qβ platform and emphasize the methodologies and medical applications associated with the system.

## 2. The Concept and Consequence of Evolution in the Medical Field

The desire of the molecular biologist to engineer natural biological entities that address future evolution are growing significantly. This includes the use of Darwin’s postulates as they relate to in vitro molecular evolution.

The major success of in vitro evolution is best achieved with a system bearing a direct linkage between the genetic information and the trait or outlook representing the dichotomy prone by Ronald Fisher and Gregor Mendel [13]. The RNA phage is appropriate for evolutionary study with its single genome (genotype) and shell protein (phenotype), which facilitate variation of information and persistence or extinction of phages respectively. The phage stands in contrast to prokaryotic and unicellular eukaryotic organisms that are composed of far more complex synthetic pathways, which each can be subjected to individual evolutionary study [13,14,15]. Moreover, the biological time frame for most studies in a complex system can be very long. The existence of a clear linkage between genotype and phenotype makes it easier for in vitro study as is the case of RNA phage display method proposed.

In a quest to give insight to the concern of RNA virus replication in a host cellular DNA replication system, Sol Spiegelman and his colleagues presented the first in vitro Darwinian evolution experiment. RNA virus replication, particularly RNA phage was found to be the perfect model system for evolutionary investigation [16]. The life cycle of RNA virus was extremely short in time with a very high number of progenies per cycle making them the perfect candidates for this kind of work. Moreover, the genetic variation processes between offspring and parent were observed and due to occasional errors. The errors were found to be the result of enzymatic machinery that lacks the proofreading repair activities during replication, explaining the difference between parent and offspring [17,18]. One of the major consequences of the lack of correction of errors during RNA replication is the existence of viruses as a complex population with a dynamic distribution of related genomes called quasispecies, by Manfred Eigen. The theory of Eigen shows that the target of selection includes not just the fastest growing replicator (replicon), but also a spectrum of mutants, which are evaluated as a single entity, the “quasispecies” and a model system of the first form of life on earth [13].

In this theory of quasispecies, the living RNA molecules are in equilibrium, which involved a master genetic sequence and other closely related copies. The master sequence always represents the important portion (in concentration) of all favored sequences and not just the consensus sequence although it may represent it sometime. To be claimed perfect, a copy has to be much closer to the master. For RNA viruses, the term population was assigned to wild type and not limited to a single genome with defined sequence [14]. The step from the RNA replication to the live virus shows the persistence in the environment of the surviving population, meaning mutation can be the consequence of environmental factors [15]. The permanent interaction between viral population and the environment can generate long-term variants or survival quasispecies, which can be subjected to continuous mutation and competition for resources [16] and survive (“Survival of fittest” of Darwin). Like in all RNA virus cases and our developed RNA phage display system, the genetic variation rate during replication is the conceptual point (pivotal) of the novel technology.

The fact that RNA populations continuously vary during replication make them more adaptive than their DNA counterpart and/or organisms. The variation rate increases with the reduced genome size [17]. The adaptation potential of RNA viruses (RNA phages in particular) can be exploited for in vitro evolutionary biotechnology. Recently, plasmid variants and derivatives with the full cDNA of the RNA coliphage Qβ were obtained with plasmid pBR322 [18], which produced Qβ phage after being transformed into *Escherichia coli* HB101 The subsequent construction of the vector could be used to insert foreign functional protein genes. The stable recombinant plasmid would be expressed in order to display a pool (library) of hybrid phages exposing the foreign proteins on the phage surface. The most adapted variant phages can then be concentrated and selected by panning which is an artificial selection against a known target [19]. In our recently developed biopanning, parameters of the natural pressure can be mimicked and adjusted, therefore generating the most adapted variants important for this in vitro evolution work.

## 3. RNA-Coliphage Qβ Display

**Figure 1:**
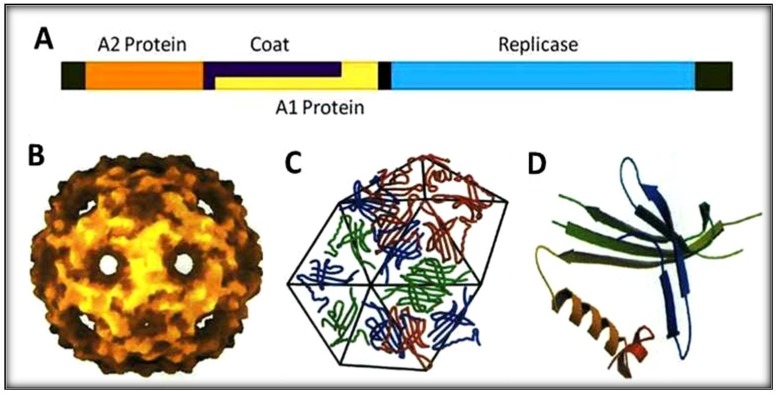
Schematic representation of RNA-coliphage Qβ. (**A**) The genomic organization with four protein genes (1 to 4220 bases). (**B**) Icosahedral shell of the phage. (**C**) Capsomers making hexamers geometry. (**D**) Coat protein structure with five-stranded antiparallel and hairpin.

The phage display technique has mostly been applied using the M13 phage which contains a single-stranded circular DNA genome with 6407 nucleotides [20,21]. The M13 display system is however a DNA replication system with very low mutation rates, which prevent and negatively affect a rapid evolution and adaptation of display biomolecules [22,23]. The polymerase of the RNA viruses (e.g., the replicase of Qβ) lack the proofreading activity and for this reason the mutation rates of these RNA viruses greatly exceed those of DNA counterparts [22,24,25]. Qβ RNA phages possess very attractive features including high mutation rates, high population and short replication time all of which can be exploited for this study [26,27]. Found throughout the world in bacterial isolates associated with sewage and animal feces, Qβ phages are small positive-stranded RNA viruses infecting *E. coli* [23,24]. Each infectious Qβ virion is about 25 nm in diameter. Qβ is made up of four genes within the 4220 nucleotides genome encoding the subunit II (β) of replicase, a major coat protein, a maturation protein A_2_ and the read-through or minor coat protein A_1_ [24]. The main difference between Qβ and another RNA phage in the same family, the MS2 phage, lies in the minor coat protein, which is the key target in this study [28,29]. The phage Qβ genome is surrounded by an icosahedral shell of 178 coat protein molecules (capsomers) and two additional capsomers are internal and associated with genomic RNA [26]. Each capsomer is comprised of 132 amino acids. A capsomer is made up of a five-stranded antiparallel β-sheet “core”, a hairpin and two contiguous β-helices on the outside [28,30]. These capsomers are linked together by disulfide bonds in covalent pentamers and hexamers with a stoichiometric ratio of 12:20 and a well-known crystal structure [31]. The A_1_ protein shares the same initiation codon with the coat protein and is produced during translation when the coat protein’s UGA stop codon triplet (about 400 nucleotides from the initiation) is suppressed by a low level of ribosome misincorporation of tryptophan [10,21] (Figure 1). The development and achievement of this Qβ display system was based on some features and known functions of the genome and proteins of the phage described here.

## 4. The Biology of RNA Qβ

### 4.1. The Maturation Protein or A_2_

The genome of Qβ is encapsulated by capsomers that make up the icosahedral structure [28]. A five-stranded β-sheet core, a hairpin and two helices on the outside of the particle make up the coat protein, and all form a lattice with the triangulation number T = 3 [29]. The Qβ virion contains only one copy of the 48.55 kDa maturation protein A_2_. Although the A_2_ protein is insoluble in an isolated form, it remains soluble when fused to maltose-binding protein or MBP [30,32]. Crystallography reveals a four-molecular structure, of a highly elongated shape, about 50 Å in width and 110 Å in length. Resembling a bow, half of the protein is built of four α helices while the other half contains β strands, both are located on opposite ends of the molecule. There are two distinct positive charged areas in the α-helical portion, their identified function will be uncovered below.

The A_2_, or minor constitute, is involved in pilus recognition and host cell binding [21]. It also serves as a protective aspect for the virion against external ribonucleases [21,33]. Unlike the MS2 phage, which contains a separate lysis gene, Qβ does not contain an independent lysis gene. Bernhardt and colleagues found that A_2_ blocks the synthesis of murein precursors in vivo by inhibiting MurA which is the catalyst of the committed step of murein biosynthesis [34]. The expression of MurA or induction of a *murA^–^* clone delays lysis or completely abolishes lysis respectively [34]. In a study conducted by Karnik and Billeter, overexpression of A_2_ was shown to trigger cell lysis [12]. Karnik and Billeter cloned complete and partial (mutated and non-mutated) Qβ sequences [35]. In cells containing plasmids for the full Qβ genome and A_2_, phage production increased 100-fold over cells with the Qβ genome plasmid only. Plasmids containing a mutation at the cistron of A_2_ did not promote cell lysis [32]. Their experiments also concluded that the entire A_2_ protein must be synthesized to mediate cell lysis; partial synthesis did not lead to cell lysis.

**Figure 2:**
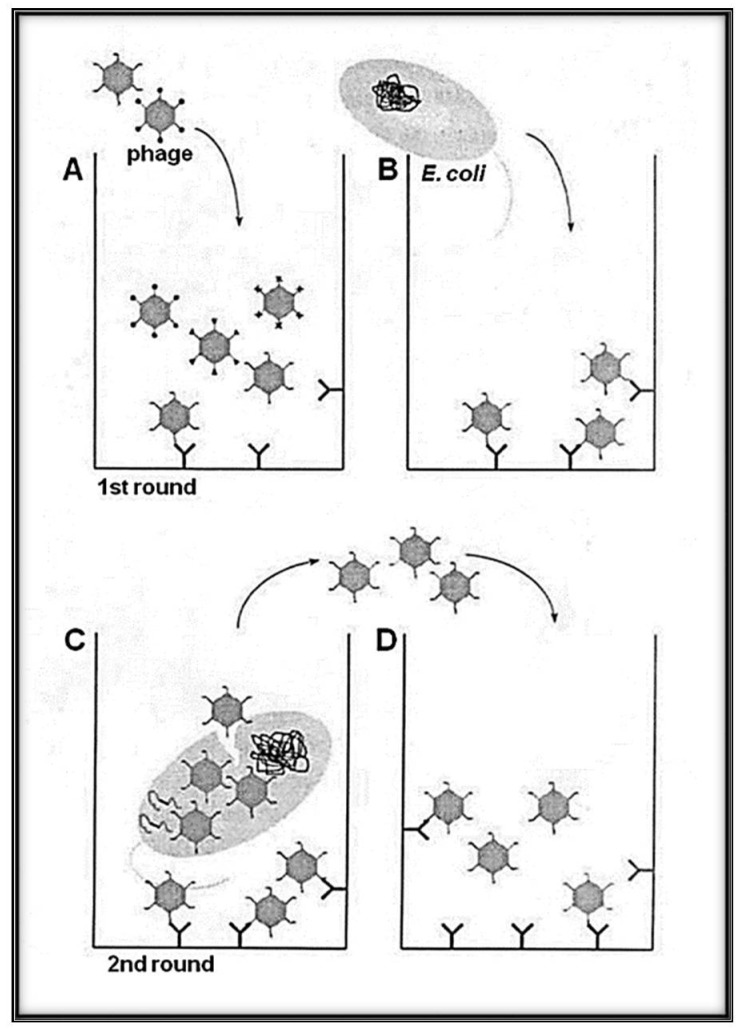
Schematic representation of the panning methodology with Qβ phage variants (library) with selection and amplification on target avoiding harsh condition (heat and/or acidic elution). (**A**) A variant of phages solution exposing the library of interest is loaded to the well of a plate pre-coated with the desired target (bio or abio). (**B**) The indicator bacteria (*Escherichia coli* Q13) at the log phase growth are added to the well to allow a round of amplification. While the phages, having low affinity to the target, are removed by series of washings, the selected candidates are amplified. (**C**) High-affinity phages bound to the target can infect *E. coli* Q13 by adsorbing to the A_2_ and injecting its RNA via the F^+^ pilus. (**D**) Phages newly obtained after indicator *E. coli* infection were transferred to new wells containing the immobilized target for the next round of biopanning. Several rounds can be used for the enrichment interactions bio-bio or bio-abio.

The maturation protein is also essential for infectivity in the Qβ infection cycle. In the conventional M13 DNA phage display, infectivity and surface expression of peptides is a characteristic held by the same minor coat protein called pIII. This gives Qβ an advantage during biopanning by not needing an acid elution step (Figure 2). In order to inject its RNA, the Qβ phage must first be adsorbed to the sex pili via the A_2_ protein [36]. As the cycle continues, while the ribosome is translating binding the coat protein region, it induces the refolding (secondary structure) of the RNA that sets free the replicase start region and the synthesis of the β subunit can commence. A minus strand is produced which acts as a template for the plus strand. During its synthesis, the ribosome binding site (RBS) of the A_2_ is accessible only for a short duration so that a small amount of the A_2_ can be produced [37,38]. This occurs due to a small relaxation state of the template before its folding into a secondary structure, closing the region. Along with the recently discovered structure, it was determined that a portion of the α-helical domain is involved in genomic RNA binding.

### 4.2. The Read-Through Protein or A_1_

The RNA bacteriophage Qβ encodes genes essential for the production of proteins such as those that compose its outer shell termed the coat and minor coat proteins. Howbeit, we will focus on the minor coat protein A_1_ protein, due to its malleability and efficacy. The A_1_ (minor coat) protein is a read-through protein and is important for the formation of infectious phage particles [39]. Translation of the coat proteins for Qβ phage initially occurs concurrently however, intermittent read-through by ribosomes of the leaky stop codon of the coat protein, allows for extension of translation beyond the C-terminus [28]. This read-through mechanism is employed by a number of viruses as a means of regulation.

The read-through domain of Qβ A_1_ protein has a 15-residue-long polyproline type II helix PPII. The A_1_ protein motif suggests its relevance and complete functionality as these PPII motifs are known to foster protein–protein and protein–nucleic acid interactions that are essential in protein assembly, signal transduction, serve as binding sites and bacterial and viral pathogenesis [29]. It was previously proposed that the probable copy number of A_1_ display sites per Qβ phage were between 3–7% in total [21]. However, it is now known that the A_1_ protein has twelve vertices that are evenly displayed across the surface of the Qβ phage [33]. A previous study has shown that small extensions, approximately 222 nucleotides long, can be fused in frame to the 3′ end of the A_1_ protein without negatively affecting the functionality or efficacy of the phages level of infectivity [33].

Although there is minimal research in the literature regarding the functionality of A_1_ protein, current studies investigating the structure of Qβ phage and its constituents indirectly suggest the potential for the exploitation of the A_1_ protein’s motif capacity for detection assays, drug delivery, and vaccines.

### 4.3. The Replicase Protein or REP

The Qβ replicase is an RNA-dependent RNA polymerase (RdRp). It differs from the reverse transcriptase found in retroviruses, which are RNA-dependent DNA polymerases (RdDp), in that it catalyzes the transcription of RNA from an RNA template, rather than RNA from a DNA template. This protein is encoded in all positive-stranded RNA viruses. The replicase is a holoenzyme consisting of four subunits: the β subunit (65 kDa) that is encoded by the phage itself, as well as three other subunits encoded for by the natural host of Qβ, *E. coli*, the α ribosomal protein (S1, 70 kDa), and two host translation elongation factors, γ (EF-Tu, 45 kDa), and δ (EF-Ts, 35 kDa). The β-subunit consists of a finger, thumb, and palm domain. The finger and thumb domains interact via hydrophobic interactions with elongation factors subunits γ and δ (EF-Tu and EF-Ts) allowing them to bind the subunit tightly [12]. This interaction between the virus and the host ensures specificity of both the template and the polymerase [40].

The S1-γ subunit is part of the bacterial 70S ribosome and is one of 21 proteins that comprises the smaller 30Ss subunit and is responsible for translation of mRNA within the cell after transcription by assisting mRNA in binding to the 30S ribosome subunit [33]. S1 has a molecular weight of 70 kDa, but has a sedimentation ratio lower than expected, due to its elongated shape [30,38]. The structure of the right-handed S1 protein is comprised of a finger, thumb, and palm domain. The finger domain consists of four antiparallel β-sheets and six α-helices. The thumb domain consists of two bundles of α-helices, comprised of three helices each as well as a three antiparallel β-sheets. The palm domain is comprised of five antiparallel β-sheets flanked by four α-helices [41].

Elongation factor subunit γ is a translation elongation factor that binds to aminoacylated tRNAs (aatRNA) and assists them in moving into the correct position within the ribosome in the A-site via the formation of a ternary complex that consists of EF-Tu:GTP:aatRNA [40]. It does this by hydrolyzing the power of GTP into GDP^+^ inorganic phosphate. It also plays a role in the accuracy of translation. After hydrolysis of GTP to GDP, the GDP-bound EF-Tu is then released from the ribosome. Elongation factor subunit δ is a second elongation factor in *E. coli* that allows EF-Tu to hydrolyze GTP into GDP. Elongation factor subunit γ then releases the bound GDP where it can be recycled and allows for EF-Tu to bind a new GTP to catalyze another aatRNA. Upon infection of the host cell by the Qβ virus, the β subunit hijacks these three subunits of the bacteria creating the replicase holoenzyme using the S1 subunit, EF-Tu, and EF-Ts as its coenzymes with the replicase becoming the β subunit, and the S1, EF-Tu, and EF-Ts becoming the α, γ, and δ subunits, respectively. It is the replicase core complex (β subunit, EF-Tu and EF-Ts) that possesses polymerization activity [40,42].

Host factor (HF1) is a 102 amino acid protein provided by the *E. coli* bacteria encoded for by the *hfq* gene, is also required for initiation of replication and initiation will not occur in the absence of the enzyme [43]. The rate of replication depends on the ratio of HF1 to RNA rather than on the ratio of RNA to replicase [20,23,38]. Once the 215 kDA replicase holoenzyme is completely assembled after hijacking the host’s S1 ribosomal protein as well as the EF-Tu and EF-Ts elongation factors, replication of Qβ’s positive-stranded genome can begin, and the genome is first synthesized into a complementary negative-stranded RNA that will serve a template for the synthesis of more positive-stranded RNA that can serve as mRNA. Both the plus and minus strands will serve as templates during replication rather than just the minus strands. This causes replication to double in each round [34]. The replicase is capable of amplifying Qβ’s genome 10,000-fold within one hour and Qβ can use both the positive and negative sense RNA strands as a template for replication; a feature that allows for exponential amplification.

Qβ’s replicase has a unique set of features that not many other replicases possess. In addition, to amplifying its genome more than 10^4^ in less than an hour, it is able to distinguish between its own RNA and a vast amount of host RNA and only replicate its own RNA, as well as do both of these things without the use of endogenous primers [34]. The template is recognized by the *S*1 protein and the EF-Tu [34]. The motif sequence required for strand recognition by Ef-Tu:EF-Ts is CCC of the CCCA sequence at the 3′ end of the template RNA [12].

Functionally, polymerization occurs once the 3′ bound end of the template enters into the initiation site of the replicase [34]. Additionally, strands are recognized by ribosomes, and minus strands are not, and must go through an alternative mechanism. The mechanism for plus strand is that it is replicated in equal amounts along with the minus strand so long as HF1 is supplied in excess. Replication proceeds linearly. Once replicase copies the plus strand, replication for that event is terminated and the strand joins a minus strand, either through template switching or it joins a free-floating minus strand. An A residue is added at the end of the free 3′ end post-transcriptionally [33].

### 4.4. Qβ Life Cycle

The Qβ phages infect bacteria with the F-pili, making them male dependent viruses. The Qβ infection cycle has been intensively investigated and simplified [44]. To start the infection during the lifecycle the phages adsorb to the specific bacterial sex pili proteins of a sensitive host cell. It is presumed that when the phages are interacting with the pili appendage via the A_2_ protein, the RNA molecules are being injected through the tubular sex pili (6–8 nm in diameter) leaving behind the empty capsid [44]. At this point of the infection the exact mechanism of the RNA genome transfer is not known. Once in the host cell the linear RNA with very high level of secondary structure (75% of the genome), is attractive to the host ribosomes. The secondary structure of Qβ RNA is mostly made of many hairpin-like loops, even at the 5′ and 3′ ends, and provides protection from host cellular exonuclease damage.

In the host cytoplasm, with its high level of RNA secondary structure, only the start region of the coat protein RNA is accessible to the ribosome. The binding of the ribosome to the start of the coat RNA region frees the replicase start gene region and allows the beginning of the early translation of this β subunit of the RNA-dependent RNA polymerase or the replicase [45]. The β subunit of the replicase requires a few more proteins from the host bacterium for its complete enzymatic activity (see above Section 4.3).

After assemblage and formation of the active replicase complex, it competes with ribosomes for the coat protein start region. This competition results in a repression of the translation due to ribosome removal. Replication occurs after the complete repression of translation in the absence of the ribosome. The minus strand of the RNA genome is the first product of this replication that serves as the template for the synthesis of the plus strand. The A_2_ start region of the newly synthesized RNA on the plus strand, not completed, would be attractive to ribosomes. The temporary availability of the A_2_ start gene to ribosomes, would produce a small amount of A_2_ proteins. The openness of the A_2_ protein gene to the ribosome is the result of a short relaxation (level free energy) of the RNA template secondary structure, which always prevents this accessibility. The plus strand produced by replication would serve as template for dual purpose: (i) replication and synthesis of progeny RNA molecules; and (ii) translation and production of more replicase enzymes and coat proteins for viral shells [46]. The further production of a large amount of the coat protein represses the replicase’s β subunit. This later repression of the replicase later in the phage cycle is done by the coat protein that binds to the RNA start of the replicase. The replicase repression induces the initiation of the coat protein shell formation and the final encapsulation of the plus strand RNA of the phage, yielding newly viable phages [47]. With the newly confirmed intimate fit between A_2_ protein and MurA, the mechanistic pathway that leads to host cell lysis during Qβ infection is now clear [48].

## 5. Medical Applications of Qβ In Vitro Evolution

Potential applications for Qβ’s replicase include usage in novel approaches to polymerase chain reaction (PCR). Isolation and use in place of traditional polymerases in PCR reactions. Unlike conventional PCR, where a short target sequence is amplified, Qβ’s replicase can mass produce using midivariant RNAs (MDV-1). Unlike most polymerases which amplify from the template strand only, Qβ’s replicase can use both the template and the product strands as templates during each round of amplification [49]. These MDVs product complements to the parent strand and in each round of infection, both sets of strands get amplified in about 20 s, generating more than a trillion copies. Additionally, the Qβ replicase does not require high temperature for denaturation as do traditional PCR enzymes such as *Thermus aquaticus* DNA polymerase (Taq). The entire process can occur at 37 °C. As mentioned, Qβ’s replicase has high specificity for its template, a fact that can be overcome by the addition of Mg^2+^ to the solution, allowing the replicase to replicate non-native templates [33].

In our research work, plasmids harboring the full cDNA of bacteriophage Qβ were used [50]. By cloning an insertion cassette at the end of *A*_1_ gene, we used bioinformatic analysis to make sure the secondary structures of the Qβ phage RNA are conserved. Specifically, the recombinant *A*_1_ gene sequence is always fused to a foreign protein DNA or cDNA, would be optimized by selecting the most adequate codons for insertion into the cassette.

**Figure 3:**
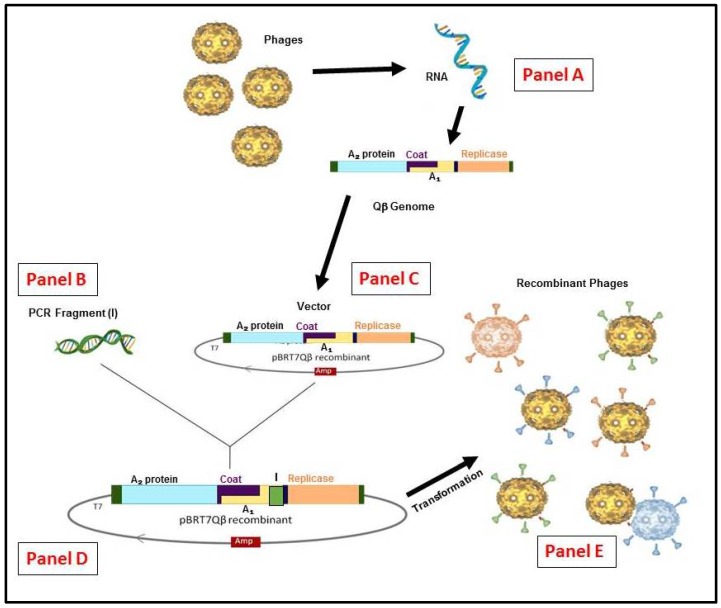
Schematic representation of the RNA Qβ phage display system methodology. (Panel **A**) The recombinant cDNA of Qβ genome or other variants can be generated by reverse transcription and polymerase chain reaction (RT-PCR). (Panel **B**) From any insert generated by (PCR) or a library constructed. (Panel **C**) The vector constructed from purified Qβ cDNA in pBR322 containing the ampicillinase gene under the control of T7 promoter [52]. (Panel **D**) the vector for display system obtained is used to insert the appropriate gene and/or library by cloning. (Panel **E**) the generation of hybrid phages or variants (if from library) after *E. coli* HB101 transformation.

Unlike DNA phages, the use of an RNA phage display technique for in vitro evolution is poorly understood. The Qβ phage is also an important tool to map the library of a virus to determine its antigenic properties which can lead to easy detection of the virus both in the field and clinical settings. Recently, we have successfully constructed and exposed a 5-mer library of FMDV VP1 G-H loop on the external surface of Qβ (Figure 3). The tandem amino acid sequence that is required for anti-FMDV monoclonal antibody SD6 was selected and evolved with our novel panning system. The tandem pair Arg–Gly was essential for the recognition and was found to be enough for this binding amongst the spectrum of variants. In that work in addition to proving the concept, the size of the inserted library and the stability of the phages obtained were the major issues. These issues were solved, and a larger size DNA was fused with a stable A_1_ protein.

A 50-mer representing the consensus sequence of the MPER region of HIV-1 was engineered and displayed of the surface of Qβ. MPER was designed for broadly neutralizing antibodies but was poorly accessible. We have demonstrated that recombinant Qβ phage framed with gp41 MPER of HIV can be used either alone or in combination with other strategies for the production and monitoring of HIV-1 gp41 MPER-specific immune responses [11]. The results obtained from the study showed that broadly neutralizing antibodies of MPER were easily recognized by all the reported epitopes (methodology in Figure 3). This reinforces that hybrid phages could be used to assess the antigenicity of specific peptides in an infected subject. The application of RNA phage technology can be ideal for a rapid detection of an infection both in animal and human [11].

Other applications include use of nanotags as a biosensor for peptide detection. Nano-tags can be used as part of the peptide library for easy identification and tracking to establish binding of recombinant phage to the right antibody. This method was very useful in maintaining the structural conformation of the peptide as shown with FMDV, HIV-1, and severe acute respiratory syndrome coronavirus or SARS-CoV [10,11]. More so, the RNA phage has been designed to bind and block breast cancer resistance protein known as P-glycoprotein [51]. This is a big breakthrough to the future of therapeutic tools against cancer using Qβ phage and nanobodies.

## 6. Conclusions

For the past hundred years, phages have provided insight into molecular biology, pathology, immunology, structural biology and medical technology. The quasispecies nature of RNA viruses has compromised numerous antiviral therapeutic agents. The RNA phage display system methodology described here is simple, low in cost and risk, and is still in its infancy and remains a powerful tool for in vitro evolution and application to the medical field. The RNA display can serve as platform for RNA virus vaccine development with its quasispecies aspect. The RNA phage can display antigens for a particular pathogen allowing it to serve as a point of care by facilitating detection and concentration of pathogen specific. This system can be further developed to play a role of biosensor for detection of disease in developing countries where sophisticated equipment is not available. Live RNA viruses are deadly and should be avoided in vaccine evaluation. Live viruses could be substituted or represented by this RNA phage display system, avoiding biohazards usually seen during live virus laboratory manipulations. With the volume of reports and papers on RNA viruses’ replication, mutation, resistance to antiviral therapy, Red Queen Hypothesis, and escaping immune molecules, the struggle and difficulties to develop vaccines against these viruses can be alleviated in the coming century using RNA phage display system.
